# Prediction of MHC class II binding peptides based on an iterative learning model

**DOI:** 10.1186/1745-7580-1-6

**Published:** 2005-12-13

**Authors:** Naveen Murugan, Yang Dai

**Affiliations:** 1 Department of Bioengineering (MC063), University of Illinois at Chicago, 851 South Morgan Street, Chicago, IL 60607, USA

## Abstract

**Background:**

Prediction of the binding ability of antigen peptides to major histocompatibility complex (MHC) class II molecules is important in vaccine development. The variable length of each binding peptide complicates this prediction. Motivated by a text mining model designed for building a classifier from labeled and unlabeled examples, we have developed an iterative supervised learning model for the prediction of MHC class II binding peptides.

**Results:**

A linear programming (LP) model was employed for the learning task at each iteration, since it is fast and can re-optimize the previous classifier when the training sets are altered. The performance of the new model has been evaluated with benchmark datasets. The outcome demonstrates that the model achieves an accuracy of prediction that is competitive compared to the advanced predictors (the Gibbs sampler and TEPITOPE). The average areas under the ROC curve obtained from one variant of our model are 0.753 and 0.715 for the original and homology reduced benchmark sets, respectively. The corresponding values are respectively 0.744 and 0.673 for the Gibbs sampler and 0.702 and 0.667 for TEPITOPE.

**Conclusion:**

The iterative learning procedure appears to be effective in prediction of MHC class II binders. It offers an alternative approach to this important predictionproblem.

## Background

Immune responses are regulated and initiated by major histocompatibility complex (MHC) molecules, which bind to short peptides from antigens and display them on the cell surface for the recognition by T cell receptors. The specificity of this binding can be predicted from the amino acid sequence of a peptide. Such predictions can be used to select epitopes for use in rational vaccine design and to increase the understanding of roles of the immune system in infectious diseases, autoimmune diseases, and cancers.

There are two types of MHC molecules, class I and class II, and both are highly polymorphic. The core binding subsequence of both MHC I and II is approximately 9 amino acids long. However, the MHC I molecules rarely bind peptides much longer than 9 amino acids, while MHC II molecules can accommodate longer peptides of 10–30 residues [1-3]. The presence of the binding core with a uniform length for MHC I molecules makes the prediction of peptide-MHC binding relatively easier. Many different methods have been developed for the prediction of peptide-MHC binding, including simple binding motifs, quantitative matrices, hidden Markov models, and artificial neural networks [4-8]. These methods can be readily applied to MHC I molecules, since the binding motif is well characterized and most of the natural peptides that bind MHC I molecules are of close to equal length.

The prediction of MHC class II binding peptides is a difficult classification problem. MHC class II molecules bind peptides that are 10–30 amino acids long with a core region of 13 amino acids containing a primary and secondary anchor residues [2,9,6,11]. Analysis of binding motifs has suggested that a core of only 9 amino acids within a peptide is essential for peptide-MHC binding. Reported binding peptides usually have variable lengths and an undetermined core region for each peptide. Therefore, a search for the binding core region can circumvent the problem of variable lengths.

Efforts have been focused on how to align the peptides such that a block of the peptides can be identified as the binding cores. The alignment of peptides is searched based on evolutionary algorithms [12], the Gibbs sampling method [13], and a recent method motivated by the ant colony search strategy [14]. The former looks for a position scoring matrix with the highest fitness score (predictive power) through the genetic operator of mutation. The latter two methods attempt to find an optimal local alignment by means of Monte Carlo Metropolis sampling in the alignment space or by the collective search strategy of ant colony systems, respectively. The binding cores with same length are identified from the alignment, and a scoring matrix used for prediction is established from these binding cores. In the work of Brusic et al. [12], the alignment of peptides is treated as a pre-processing procedure. Upon the determination of the binding cores, a binary classifier is then learned with artificial neural networks using amino acid sequences presented in the binding core as a positive training set and other non-binding peptides as a negative training set. In Nielsen et al. [13] and Karpenko et al. [14], a position scoring matrix is obtained from the best alignment and used for scoring peptides. Most of these alignment-based predictors have achieved reasonably good performances. However, a common complication involved in these methods is the correct choice of associated parameters. The tuning of the parameters could be complicate. A similar work is by Bhasin et al. who used a pre-processing procedure called MOTs to filter the putative binding core for binding peptide sequences and subsequently trained the classifier based on the support vector machine (SVM) [15] with those binding core sequences and random sequences [16]. Another method using an iterative approach has been developed based on a stepwise discriminant analysis model [17,18]. More recently a model based on Bayesian neural networks has been developed [19].

This work is motivated by a machine learning model designed for a training task with only positive and unlabeled examples in text mining. This type of training set is in evidence in various applications in which the identification of a positive training example is labor intensive and time consuming. The basic idea developed for this learning task is the use of a binary classifier to filter out positive examples from the unlabeled set and include them into the positive set through an iterative procedure [20,21]. A classifier is trained at each iteration by simply assigning positive examples the label 1 and unlabeled examples the label -1 to form normal binary training sets. A classifier can be learned by the use of different binary classification methods such as the Naïve Bayesian or support vector machines.

The unlabeled and labeled examples in the prediction of peptide-MHC binding can be introduced naturally through the encoding mechanism. A sliding window scheme with a window length of 9 is applied to binding peptides. This procedure breaks a peptide into a set of nonamers of equal length. The binding core, which is unknown, is one of the nonamers. The nonamers from all the binding peptides serve as unlabeled examples in which the positive examples, i.e., nonamers of binding cores, are included. Similarly, all nonamers obtained from the non-binding peptides serve as negative examples. It is noted that the situation in this application is opposite to that of text mining. Here a negative set and an unlabeled set containing potential positive examples are presented. However, the same strategy described previously for text mining can be applied. The approach here is to filter out non-binding nonamers in the unlabeled set iteratively. This iterative learning model enables the use of the non-binder information for the identification of the binding cores and to generate the predictor simultaneously. This is different from the three alignment based methods mentioned earlier in which the identification of binding cores relied only on binding peptides.

The linear programming (LP) model proposed by Bennett and Mangasarian [22] is used as the learning model for binary classification at each iteration. This model has several advantages over other learning methods such as support vector machines, Naïve Bayesian, and artificial neural networks. First, there are only a few parameters and they are very easy to tune. Second, a linear program can be solved very fast and it embodies favorable properties which allow sensitivity analysis. Therefore, if the subsequent linear program is only different for a small number of constraints, then the corresponding optimal solution can be found through a re-optimization procedure that uses the information of the current optimal solution. This is particularly important for the iterative learning procedure as only a small number of nonamers is removed from the positive training set at each iteration.

This model was evaluated with benchmark datasets from MHCBench against other major existing methods. The computational study demonstrates overall that this method can achieve comparable or superior performance in comparison with the competing predictors, such as the Gibbs sampler [13] and TEPITOPE [10]. The average areas under the ROC (Receiver Operating Characteristic) curve [23] obtained from one variant of our model are 0.753 and 0.715 for the original and homology reduced benchmark sets, respectively. The corresponding values are 0.744 and 0.673 for the Gibbs sampler and 0.702 and 0.667 for TEPITOPE.

## Methods

### LP model for classification

Consider a set of positive examples *x*_*i*_, *i *= 1,...,*m*_+ _and a set of negative examples *x*_*i*_, *i *= 1,...,*m*_-_, each of which is a point in an *n*-dimensional space. The LP model for a binary classification problem in (Bennett et al., 1992) is as follows.



where *y*_*i *_= 1 or -1 is the label assigned to each positive or negative example, respectively.

This model generates a separating hyperplane with the smallest amount of misclassification error. It has been proved that this linear program always returns a non-trivial solution of *w*, which permits a linear classification function, even in a non-linear separable case [22]. The decision function, denoted by *f*(*x*) = *w*^*T*^*x*+*b*, assigns a label to an example *x *by the sign of *f*(*x*).

### LP model for MHC class II problem

**Figure 1:**
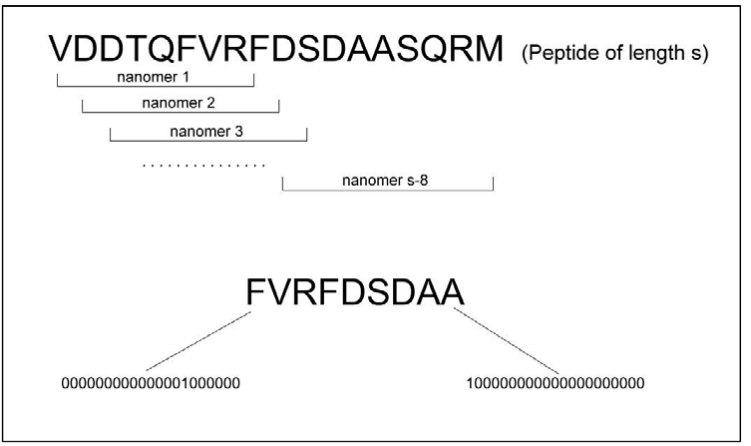
Top: A peptide has been reduced to a set of nonamers. Bottom: A nonamer is encoded as a 180-dimensional vector.

A set of nonamers can be obtained by sliding a window of length 9 along each MHC class II binding peptide as described previously. A peptide of length *s *will have *s *- 8 nonamers (see top panel, Figure 1). These nonamers are further reduced to a set of putative nonamers based on the knowledge that the residue in the first position of the nonamer has to be hydrophobic in order for it to bind to an HLA-DR MHC II molecule. This set of putative nonamers is considered as an unlabeled set. Each nonamer in the unlabeled set is assigned the label 1 temporarily.

The negative set of nonamers can be obtained similarly from the non-binding peptides. Each nonamer in this set is assigned the label -1. All redundant nonamers in both sets are removed. The remaining nonamers are subject to further preprocessing steps, which will be described later.

An amino acid at each position of the nonamer can be encoded by a 20-dimensional vector. Each coordinate of the vector is either 1 or 0, representing the presence or the absence of a particular amino acid. Accordingly, each nonamer can then be represented as a 180-dimensional vector (see bottom panel, Figure 1).

Assume that there are *m*_+ _binding and *m*_- _non-binding peptides. Each encoding vector of a nonamer for a peptide *i *is denoted by . Assume that each binder *i *permits *i*_*k *_putative nonamers. By using the LP model given before, our problem can be formulated as the following linear program:



where *C*_1 _and *C*_2 _are coefficients that will be determined through cross-validation on the training set. Note that we have extended the LP model (1) by allowing the change of coefficients *C*_1 _and *C*_2 _associated with the error terms in the objective function in LP (1). This extension aims at the control of the weights on error terms so that some non-core nonamers in the positive set are deliberately misclassified. This is a chief characteristic of our learning model.

### Iterative procedure

The iterative training process consists of the following major steps. First, a weight vector *w *and value *b *are obtained by solving the LP (2) for fixed *C*_1 _and *C*_2_. This solution is used to score each nonamer in the positive training set based on the function *f*(*x*) = *w*^*T*^*x*+*b*. Nonamers with negative scores from the positive set are moved to the negative set. Subsequently, the LP is solved for the altered training sets. This process is repeated for a number of iterations, which will be determined through cross-validation (CV). The function *f*(*x*)defined with the final LP solution *w*, and *b *is used for the prediction of peptides in the testing set. A peptide that has at least one positively scored nonamer is considered as a binder; and otherwise it is considered a non-binder. If several nonamers from one peptide have a positive score, then the nonamer with the highest score is considered as the binding core for that peptide. Note that there may be no binding core identified for certain binding peptides in the final positive training set.

In addition to the learning model described above, two other variants were considered. In the first variation, the nonamers in the positive set evaluated with a negative score were discarded instead of being appended to the negative set at each iteration, as these nonamers may not necessarily be true non-binders. In the second variation, at most two nonamers with the highest positive scores from each peptide were allowed to remain in the positive set and the remaining was discarded. The approach in this variant of the LP is motivated by the observation that the binding core is likely to occur among the high scoring nonamers for each peptide. (From our preliminary study on peptides from the training set with known binding core regions, it was observed that there was no significant improvement in performance from using the top three or four nonamers over the top two nonamers.)

These variants of the LP method are referred to as LP_append, LP_discard, and LP_top2 in the discussions below. For LP_append, LP_discard, the number of iterations for which the LP process is repeated and the coefficients *C*_1 _and *C*_2 _were determined by a 5-fold CV on the training set. For LP_top2, the CV procedure only determines the coefficients *C*_1 _and *C*_2_, since LP_top2 terminates after the second iteration. The area under the ROC curve was the criterion for the evaluation of predictors. The final predictor for each method was obtained by training the whole training set with the optimal parameters determined from the 5-fold CV. The linear programming package GLPK [24] was used to solve the LP given (2).

## Data sets

### Training data sets for HLA-DR4 (B1*0401) allele

The sequences of peptides binding to the MHC class II molecule HLA-DR4 (B1*0401) from the SYFPEITHI [6] and MHCPEP [12] databases were extracted. Since the SYFPEITHI database has more peptides now than in 1999, peptides sequences added to the database after year 1999 were eliminated to make it comparable to the dataset used in Nielsen et al. [13]. This set consists of 462 unique binding peptide sequences. Non-binders for the MHC class II molecule HLA-DR4 (B1*0401) were extracted from the MHCBN database [25]. This set consists of 177 unique non-binding peptides sequences.

The binding peptides that do not possess a hydrophobic residue (I, L, M, F, W, Y, V) at the first position in putative binding cores were removed [12]. That is, a peptide was removed if no hydrophobic residues are present at the first *n*-*s*+1 positions, where *n *is the peptide length and *s *is the length of the sliding window. The hydrophobic filter removed 27 peptides. Furthermore, the set was reduced by removing unnatural peptide sequences with an extreme amino acid content of more than 75% alanine. Thus, the pre-processing procedure gives 462 unique binding peptides and 177 unique non-binding peptides. The length distribution in the training set ranges from 9 to 30 residues, with the majority of peptides having a length of 13 amino acids. These peptide sequences were then used to obtain nonamers with the sliding window scheme described earlier. All redundant nonamers and nonamers that do not have a hydrophobic residue at position 1 were removed. The final numbers of nonamers obtained from the binding and non-binding peptides are 796 and 903, respectively.

### Testing data sets for HLA-DR4 (B1*0401) allele

Ten benchmark datasets used in Nielsen [13] were considered in our study. These 10 datasets consist of the 8 datasets described in MHCBench [26] and 2 datasets described by Southwood [27] and Geluk [28]. The same procedure presented in Nielsen et al for the determination of binders and non-binders was followed in our study. More specifically, for the 8 MHCBench datasets, peptides with an associated binding value of zero were considered be non-binders, and all other peptides were binders. For the datasets of Southwood and Geluk, an affinity of 1000 nM was taken as the threshold for peptide binding [27]. In order to reduce the chance of over-prediction, the benchmarking was also performed on the homology-reduced datasets. The homology reduction was carried out so that no peptide in the evaluation sets had a match in the training set with sequence identity >90% over an alignment length of at least nine amino acids. Table 1 shows a summary of the original and the homology-reduced benchmark datasets, respectively. Note that there is small discrepancy in the numbers in some of the reduced sets compared with the ones reported in Nielsen [13] (From the email communication with Dr. Nielsen, there was an error in reporting the numbers in the table in their paper; however, the results on prediction presented there were based on the numbers shown in Table 1).

**Table 1:** Description of HLA-DR4 (B1*0401) benchmark datasets.

**Set**	**Original Dataset**			**Homology Reduced Dataset**		
	
	**Total**	**Binders**	**Non Binders**	**Total**	**Binders**	**Non Binders**
Set 1	1017	694	323	531	248	283
Set 2	673	381	292	416	161	255
Set 3a	590	373	217	355	151	204
Set 3b	495	279	216	325	128	197
Set 4a	646	323	323	403	120	283
Set 4b	584	292	292	375	120	255
Set 5a	117	70	47	110	65	45
Set 5b	85	48	37	84	47	37
Southwood	105	22	83	99	19	80
Geluk	22	16	6	21	15	6

### Data sets of HLA-DRB1*0101 and HLA-DRB1*0301 for cross-validation test

Two other datasets for the MHC class II molecules HLA-DRB1*0101 and HLA-DRB1*0301 were obtained from the MHCBN database [25]. The dataset for HLA-DRB1*0101 consists of 475 binder and 105 non-binder peptides. The dataset for HLA-DRB1*0301 consists of 219 binder and 150 non-binder peptides. The same pre-processing procedure described earlier was applied to these two sets.

## Results

### Testing of the benchmark data for HLA-DR4(B1*0401)

**Figure 2:**
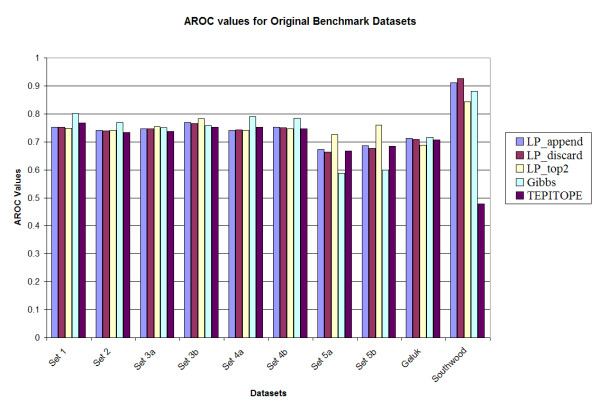
Prediction accuracy of the various methods on the original benchmark datasets.

**Figure 3:**
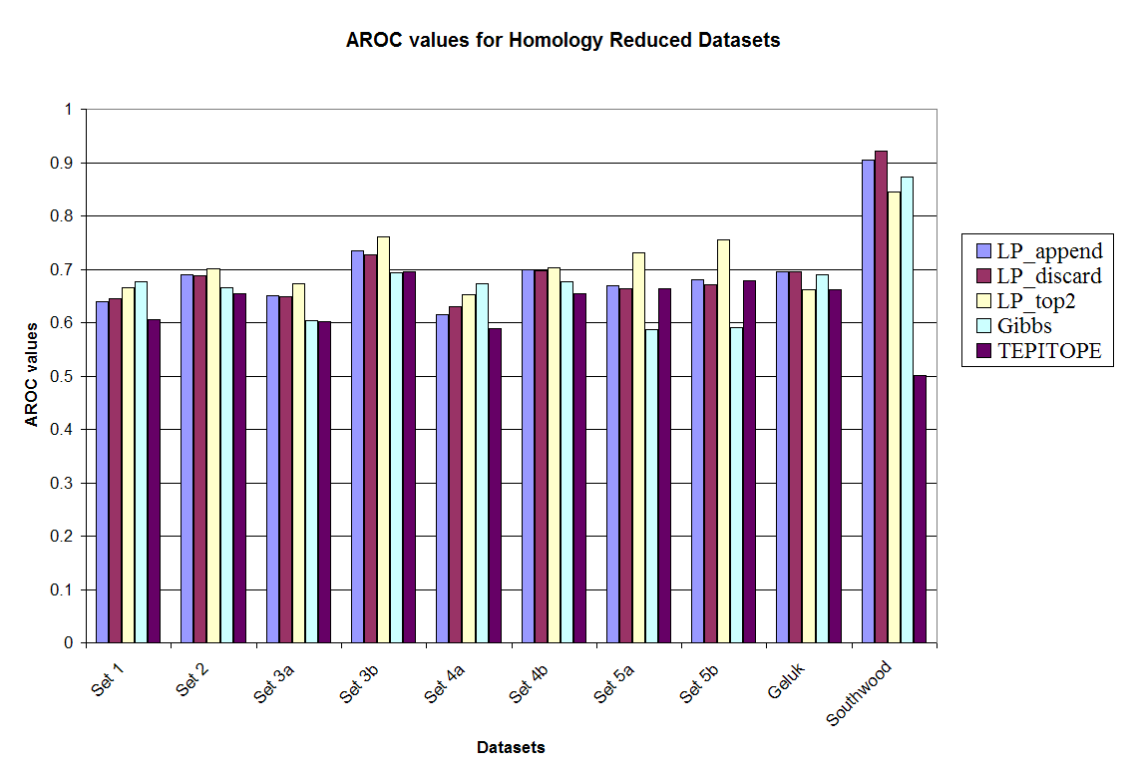
Prediction accuracy of the various methods on the homology reduced datasets.

The results of the three methods on the benchmark datasets are compared with those obtained from the Gibbs sampling technique [13] and TEPITOPE [10]. The results of the Gibbs sampler were calculated with the scoring matrix provided by Dr. Nielsen; and the results of TEPITOPE were obtained with the use of the scoring matrix from ProPred [29], which is based on the one from TEPITOPE. The performance, evaluated by the area under the ROC curve (Aroc), of each method on the 10 benchmark datasets is presented in Figure 2 and Figure 3. Table 2 gives the performance of the methods averaged over the 10 benchmark datasets. It is observed that among the three proposed methods, LP_top2 has a slightly higher average Aroc value than those obtained from the other two variants. It is also observed that all the three LP variants have higher Aroc values compared to the Gibbs sampler and TEPITOPE.

**Table 2:** The average Aroc values for different methods.

Method	Average Aroc values for the benchmark datasets	
	
	Original	Homology Reduced
LP_append	0.749	0.698
LP_discard	0.748	0.699
LP_top2	**0.753**	**0.715**
Gibbs method	0.744	0.673
TEPITOPE (Propred)	0.702	0.667

A notable observation is that the performance of the Gibbs sampler appears to deteriorate for set 5A (0.588) and set 5B (0.600), whereas the LP methods maintain the performance for those two datasets, e.g., LP_top2 has Aroc values of 0.725 and 0.760 for the original benchmark sets 5a and 5b respectively. These two datasets have higher cysteine content as compared to the training set. However, since the LP methods use both binders and non-binders to train the classifier (unlike most of the other methods in which only binders are used for training), the LP methods are more robust in performance. In addition, upon testing the method by substituting all occurrences of cysteine in all the sets by alanine [13], it was observed that the LP_top2 method obtained Aroc values of 0.815 and 0.859 for the original benchmark sets 5a and 5b, respectively; while the Gibbs sampler obtained Aroc values of 0.621 and 0.661, respectively. The details of the results are provided in Tables S1, S2, S3 and S4 in the supplementary document (see Additional files add1.doc – add4.doc). Also TEPITOPE had a poor performance for the Southwood dataset (Aroc values 0.703 and 0.630 for the original and homology datasets) due to a biased amino acid composition at position P1 and that if a modified TEPITOPE matrix at the P1 position was used, TEPITOPE could increase Aroc values to 0.786 and 0.794 for the original and homology reduced Southwood datasets, respectively [13]. For the other benchmark datasets, the performance of the modified TEPITOPE is similar to the original TEPITOPE matrix.

In order to investigate the statistical significance of the results, 1000 datasets were generated by random sampling N data points with replacement for each dataset. Here, *N *is the number of data points in the original dataset. The performance of the different methods was evaluated for each of the original and homology reduced datasets. It was observed that among the LP variants, LP_top2 had a slightly improved performance when compared to LP_append and LP_discard methods. However there was no significant difference observed in their performance. The overall average performance of the methods for the sampled datasets was also not very different from that for the original and homology reduced datasets. The details are provided in Tables S1 and S2 in the supplementary document (see Additional files add5.doc – add6.doc)

For comparison with the Gibbs sampler, the *p*-value for the hypothesis that the Gibbs method performs better than the LP method is estimated as the fraction of experiments where the Gibbs sampler has a better performance. LP_top2 was selected in this comparison. It was observed that for the original benchmark datasets, for 7 of the 10 datasets (sets 1, 2, 3a, 3b, 4a, 5a and 5b), LP_top2 performed better than the Gibbs sampling method (*p *< 0.05). For the remaining 3 datasets, there was no significant difference in performance (0.05 <*p *< 0.95). In case of the homology reduced datasets, for 8 of the 10 datasets (sets 1, 2, 3a, 3b, 4a, 4b, 5a and 5b), LP_top2 performed better than the Gibbs sampling method (*p *< 0.05). For the remaining 2 datasets there was no significant difference in performance (0.05 <*p *< 0.95). The same comparison was made between LP_top2 and TEPITOPE. It was observed that for the original benchmark datasets, for 2 of the 10 datasets (sets 5b and Southwood), LP_top2 performed better than the TEPITOPE sampling method (*p *< 0.05). For the remaining 8 datasets, there was no significant difference in performance (0.05 <*p *< 0.95). In case of the homology reduced datasets, for 7 of the 10 datasets (sets 1, 2, 3a, 3b, 4a, 5a and Southwood), LP_top2 performed better than TEPITOPE (*p *< 0.05). For the remaining 3 datasets there was no significant difference in performance (0.05 <*p *< 0.95). Details are given in Table S3 in the supplementary document (see Additional files add7.doc).

### Results for cross-validation

The LP method (LP_top2) was also evaluated using a 5-fold cross-validation for the datasets of HLA-DRB1*0101 and HLA-DRB1*0301. The results were compared against those obtained from TEPITOPE (see Table 3). The TEPITOPE matrix was downloaded from ProPred [29], and was used on the testing folds. The LP method produced Aroc values 0.779 for HLA-DRB1*0101 data set and 0.721 for HLA-DRB1*0301 dataset. The corresponding values generated from TEPITOPE are 0.842 and 0.585, respectively. The LP method appears to be more consistent in performance over different alleles.

**Table 3:** The average Aroc values from 5-fold cross validations.

Method	HLA-DRB1*0101	HLA-DRB1*0301
LP_top2	0.779	0.721
TEPITOPE (Propred)	0.842	0.585

### Prediction of binding core

The predictive ability of the LP method (LP_append) for the identification of binding cores in binding peptides was assessed for the HLA-DR4 (B1*0401) allele. The 68 peptide sequences which have information on experimentally determined binding cores, contained in the SYFPEITHI database were used for the verification. Nonamers in the initial set of putative binding cores for the HLA-DR4(B1*0401) allele that are identical to any binding cores in the 68 binding peptides were removed. It resulted in a new training set of 755 binding nonamers. The same negative nonamer set for the HLA-DR4(B1*0401) allele was used. The classifier was trained with the use of the previously described procedure. Among the 68 binding peptides, Fifty one binders which produce distinct binding cores were selected from the 68 binders. However, 6 of those had cores with a length less than 9 amino acids. After the removal of these exceptions, 45 peptides were left for the testing.

**Figure 4:**
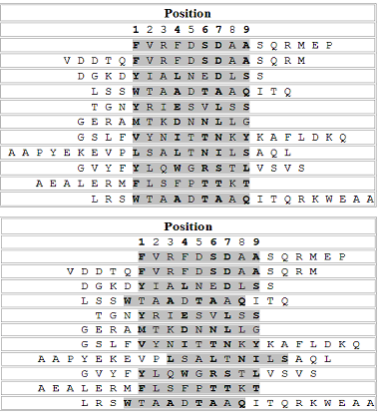
Top: The alignment of actual binding cores (shadowed) from SYFPEITHI database. Bottom: The alignment of the predicted binding cores by the LP method.

The predicted binding core is considered as the nonamer with the highest score. The numbers of identified binding cores that were within two positions from the exact binding core by the LP method, TEPITOPE, and the Gibbs sampler are respectively 41, 43, and 42. That is, each identified binding core shares at least 7 consecutive residues with the reported cores. The reason for verifying the predicted core with a shift of a few positions of the reported binding core is because that the binding affinity is not completely determined by the binding core and the flanking amino acids on both sides of the real core may contribute to the binding affinity and stability [19,30,31]. It should be noted that the Nielsen matrix used was obtained from the original training set, which includes those 68 binders. It appears that the three methods performed almost the same. The core alignment of 11 peptides out of the 45 testing peptides obtained from the LP method and the original core alignment from the SYFPEITHI database are presented in Figure 4.

## Discussion

It is important to note that the Gibbs sampler involves a set of parameters that need to be optimized using a complicated procedure before the training, whereas the LP method is very simple and the only parameters that need to be determined are the coefficients for the misclassification errors and the number of iterations. Both these parameters are easily and very quickly determined through cross-validation. This process involves no modification when applied to peptide sequences respect to MHC alleles.

A similar iterative approach for predicting HLA DR1 alleles using a stepwise discriminant analysis (SDA) has been reported [17,18]. This approach trains a linear discriminant function at each iteration and uses it to evaluate nonamers obtained from the original binding peptide sequences. Those nonamers passed the prediction threshold forms the positive training set in the next iteration. Therefore, the positive training set is dynamically changing over iteration to iteration. The negative training set remains unchanged. In this sense, Mallios' method is similar to our LP_discard or LP_top2. The discriminative features are selected based on the F-statistic from a one-way analysis of variance. The Mallios model is essentially a multiple linear regression which minimizes the sum of squared errors, while our model minimizes a weighted sum of errors.

In a recent work, a Bayesian neural network [19] was used for the prediction of MHC class II peptide binding. They concluded that their method outperforms the neural network model [12] and the SVM model [16]. Since their datasets were not available, a direct comparison could not be performed.

## Conclusion

An iterative supervised learning model has been developed for the prediction of peptide binding to MHC class II molecules. This approach was motivated by a model for building a classifier with the positive and unlabeled training sets in text mining. The major feature of this method is its iterative extracting of binding core nonamers. The iterative training process functions like an 'adaptive loop', feeding back useful information by validating against the training data. The results indicate that the performance of the new method for HLA-DR4 (B1*0401) allele is competitive to other methods. Furthermore, the method can incorporate new peptides into the training data easily. This feature makes the method far more adaptive. It is expected that the predictive accuracy will be improved, if the information on other key anchor positions is incorporated [13] and a support vector machine learning model is adapted.

## Supplementary Material

Additional File 1 This file includes Table S1 – The average of Aroc values and standard deviation for the 1000 random sampling datasets on the original benchmark datasets. Click here for file

Additional File 2 This file includes Table S2 – The average of Aroc value and standard deviations for the 1000 random sampling datasets on the homology reduced benchmark datasets. Click here for file

Additional File 3 This file includes Table S3 – P values for the statistical tests. Click here for file

Additional File 4 This file includes Table S4 – The Aroc values for the original benchmark datasets. Click here for file

Additional File 5 This file includes Table S5 – The Aroc values for the reduced benchmark datasets. Click here for file

Additional File 6 This file includes Table S6 – The Aroc values for the original benchmark datasets (Cysteine substituted). Click here for file

Additional File 7 This file includes Table S7 – The Aroc values for the reduced benchmark datasets (Cysteine substituted). Click here for file
