# Supplementary material for: Prediction of MHC class II binding peptides based on an iterative learning model
Source: Immunome Res. 2005 Dec 13;1:6. doi: 10.1186/1745-7580-1-6 (PMC1325229; doi:10.1186/1745-7580-1-6)
Supplement: Additional File 2 — This file includes Table S2 – The average of Aroc value and standard deviations for the 1000 random sampling datasets on the homology reduced benchmark datasets. [file 1745-7580-1-6-S2.doc]

Table S2. The Aroc values for the reduced benchmark datasets.

| **Reduced Dataset** | **LP_append** | **LP_discard** | **LP_top2** | **Gibbs** | **TEPITOPE** |
| --- | --- | --- | --- | --- | --- |
| Set 1 | 0.639 | 0.645 | 0.666 | 0.676 | 0.605 |
| Set 2 | 0.689 | 0.687 | 0.702 | 0.666 | 0.654 |
| Set 3a | 0.651 | 0.649 | 0.672 | 0.605 | 0.602 |
| Set 3b | 0.735 | 0.728 | 0.761 | 0.694 | 0.696 |
| Set 4a | 0.615 | 0.630 | 0.653 | 0.674 | 0.588 |
| Set 4b | 0.699 | 0.697 | 0.702 | 0.677 | 0.654 |
| Set 5a | 0.669 | 0.664 | 0.732 | 0.587 | 0.663 |
| Set 5b | 0.681 | 0.671 | 0.756 | 0.591 | 0.678 |
| Geluk | 0.696 | 0.695 | 0.662 | 0.689 | 0.662 |
| Southwood | 0.906 | 0.922 | 0.844 | 0.872 | 0.500 |
| Average | **0.698** | **0.699** | **0.715** | **0.673** | **0.630** |
