# Supplementary material for: Prediction of MHC class II binding peptides based on an iterative learning model
Source: Immunome Res. 2005 Dec 13;1:6. doi: 10.1186/1745-7580-1-6 (PMC1325229; doi:10.1186/1745-7580-1-6)
Supplement: Additional File 3 — This file includes Table S3 – P values for the statistical tests. [file 1745-7580-1-6-S3.doc]

Table S3. The Aroc values for the original benchmark datasets (Cysteine substituted)

| **Original Dataset** | **LP_append** | **LP_discard** | **LP_top2** | **Gibbs** | **TEPITOPE** |
| --- | --- | --- | --- | --- | --- |
| Set 1 | 0.740 | 0.740 | 0.725 | 0.803 | 0.776 |
| Set 2 | 0.731 | 0.732 | 0.721 | 0.775 | 0.740 |
| Set 3a | 0.734 | 0.736 | 0.728 | 0.750 | 0.740 |
| Set 3b | 0.755 | 0.756 | 0.753 | 0.762 | 0.754 |
| Set 4a | 0.731 | 0.731 | 0.719 | 0.793 | 0.763 |
| Set 4b | 0.742 | 0.742 | 0.728 | 0.787 | 0.750 |
| Set 5a | 0.714 | 0.691 | 0.815 | 0.621 | 0.651 |
| Set 5b | 0.761 | 0.760 | 0.859 | 0.661 | 0.661 |
| Geluk | 0.696 | 0.688 | 0.639 | 0.705 | 0.719 |
| Southwood | 0.911 | 0.937 | 0.833 | 0.880 | 0.484 |
| Average | **0.752** | **0.751** | **0.752** | **0.754** | **0.704** |
