# Supplementary material for: Prediction of MHC class II binding peptides based on an iterative learning model
Source: Immunome Res. 2005 Dec 13;1:6. doi: 10.1186/1745-7580-1-6 (PMC1325229; doi:10.1186/1745-7580-1-6)
Supplement: Additional File 4 — This file includes Table S4 – The Aroc values for the original benchmark datasets. [file 1745-7580-1-6-S4.doc]

Table S4. The Aroc values for the reduced benchmark datasets (Cysteine substituted)

| **Reduced Dataset** | **LP_append** | **LP_discard** | **LP_top2** | **Gibbs** | **TEPITOPE** |
| --- | --- | --- | --- | --- | --- |
| Set 1 | 0.630 | 0.639 | 0.649 | 0.686 | 0.611 |
| Set 2 | 0.685 | 0.689 | 0.690 | 0.683 | 0.654 |
| Set 3a | 0.640 | 0.645 | 0.652 | 0.611 | 0.597 |
| Set 3b | 0.719 | 0.722 | 0.733 | 0.704 | 0.690 |
| Set 4a | 0.611 | 0.625 | 0.641 | 0.687 | 0.601 |
| Set 4b | 0.697 | 0.703 | 0.699 | 0.691 | 0.648 |
| Set 5a | 0.715 | 0.697 | 0.825 | 0.624 | 0.647 |
| Set 5b | 0.756 | 0.755 | 0.856 | 0.654 | 0.654 |
| Geluk | 0.676 | 0.670 | 0.606 | 0.676 | 0.676 |
| Southwood | 0.906 | 0.933 | 0.833 | 0.872 | 0.506 |
| Average | **0.703** | **0.708** | **0.719** | **0.689** | **0.628** |
