# Supplementary material for: Prediction of MHC class II binding peptides based on an iterative learning model
Source: Immunome Res. 2005 Dec 13;1:6. doi: 10.1186/1745-7580-1-6 (PMC1325229; doi:10.1186/1745-7580-1-6)
Supplement: Additional File 5 — This file includes Table S5 – The Aroc values for the reduced benchmark datasets. [file 1745-7580-1-6-S5.doc]

Table S5. The average of Aroc values and standard deviation for the 1000 random sampling datasets on the original benchmark datasets.

| **Original**  **dataset** | **LP_top2** | | **LP_append** | | **LP_discard** | | **Gibbs** | | **TEPITOPE** | |
| --- | --- | --- | --- | --- | --- | --- | --- | --- | --- | --- |
|
|  | **AVG** | **SD** | **AVG** | **SD** | **AVG** | **SD** | **AVG** | **SD** | **AVG** | **SD** |
| **set 1** | 0.749 | 0.018 | 0.753 | 0.018 | 0.753 | 0.018 | 0.712 | 0.021 | 0.767 | 0.015 |
| **set 2** | 0.741 | 0.021 | 0.742 | 0.021 | 0.740 | 0.021 | 0.698 | 0.022 | 0.733 | 0.019 |
| **set 3a** | 0.753 | 0.023 | 0.747 | 0.023 | 0.747 | 0.023 | 0.684 | 0.026 | 0.736 | 0.022 |
| **set 3b** | 0.783 | 0.022 | 0.772 | 0.021 | 0.768 | 0.022 | 0.699 | 0.026 | 0.754 | 0.021 |
| **set 4a** | 0.741 | 0.020 | 0.741 | 0.020 | 0.744 | 0.020 | 0.702 | 0.023 | 0.752 | 0.018 |
| **set 4b** | 0.748 | 0.021 | 0.753 | 0.021 | 0.751 | 0.021 | 0.713 | 0.023 | 0.747 | 0.020 |
| **set 5a** | 0.726 | 0.046 | 0.673 | 0.049 | 0.665 | 0.049 | 0.555 | 0.058 | 0.669 | 0.048 |
| **set 5b** | 0.756 | 0.054 | 0.682 | 0.058 | 0.673 | 0.058 | 0.592 | 0.063 | 0.682 | 0.058 |
| **geluk** | 0.690 | 0.053 | 0.714 | 0.049 | 0.709 | 0.050 | 0.580 | 0.053 | 0.705 | 0.060 |
| **southwood** | 0.842 | 0.099 | 0.911 | 0.092 | 0.925 | 0.087 | 0.873 | 0.084 | 0.470 | 0.144 |
| **AVG** | **0.753** | **0.038** | **0.749** | **0.037** | **0.748** | **0.037** | **0.681** | **0.040** | **0.702** | **0.043** |
