# Supplementary material for: Prediction of MHC class II binding peptides based on an iterative learning model
Source: Immunome Res. 2005 Dec 13;1:6. doi: 10.1186/1745-7580-1-6 (PMC1325229; doi:10.1186/1745-7580-1-6)
Supplement: Additional File 6 — This file includes Table S6 – The Aroc values for the original benchmark datasets (Cysteine substituted). [file 1745-7580-1-6-S6.doc]

Table S6. The average of Aroc value and standard deviations for the 1000 random sampling datasets on the homology reduced benchmark datasets.

| **Reduced dataset** | **LP_top2** | | **LP_append** | | **LP_discard** | | **Gibbs** | | **TEPITOPE** | |
| --- | --- | --- | --- | --- | --- | --- | --- | --- | --- | --- |
|  | **AVG** | **SD** | **AVG** | **SD** | **AVG** | **SD** | **AVG** | **SD** | **AVG** | **SD** |
| **set 1** | 0.667 | 0.023 | 0.639 | 0.024 | 0.645 | 0.024 | 0.611 | 0.026 | 0.605 | 0.025 |
| **set 2** | 0.702 | 0.026 | 0.689 | 0.027 | 0.687 | 0.027 | 0.622 | 0.028 | 0.654 | 0.028 |
| **set 3a** | 0.673 | 0.032 | 0.652 | 0.032 | 0.651 | 0.032 | 0.566 | 0.033 | 0.602 | 0.032 |
| **set 3b** | 0.759 | 0.028 | 0.733 | 0.029 | 0.727 | 0.029 | 0.649 | 0.031 | 0.694 | 0.030 |
| **set 4a** | 0.653 | 0.030 | 0.616 | 0.031 | 0.631 | 0.031 | 0.610 | 0.031 | 0.587 | 0.032 |
| **set 4b** | 0.702 | 0.028 | 0.699 | 0.028 | 0.698 | 0.028 | 0.632 | 0.030 | 0.653 | 0.030 |
| **set 5a** | 0.734 | 0.048 | 0.671 | 0.052 | 0.666 | 0.051 | 0.564 | 0.060 | 0.664 | 0.053 |
| **set 5b** | 0.755 | 0.054 | 0.683 | 0.059 | 0.673 | 0.060 | 0.587 | 0.065 | 0.678 | 0.059 |
| **geluk** | 0.661 | 0.056 | 0.693 | 0.054 | 0.693 | 0.053 | 0.565 | 0.055 | 0.662 | 0.063 |
| **southwood** | 0.849 | 0.092 | 0.906 | 0.087 | 0.922 | 0.082 | 0.875 | 0.082 | 0.498 | 0.146 |
| **AVG** | **0.716** | **0.042** | **0.698** | **0.042** | **0.699** | **0.042** | **0.628** | **0.044** | **0.630** | **0.050** |
