# Supplementary material for: Prediction of MHC class II binding peptides based on an iterative learning model
Source: Immunome Res. 2005 Dec 13;1:6. doi: 10.1186/1745-7580-1-6 (PMC1325229; doi:10.1186/1745-7580-1-6)
Supplement: Additional File 7 — This file includes Table S7 – The Aroc values for the reduced benchmark datasets (Cysteine substituted). [file 1745-7580-1-6-S7.doc]

Table S7. P values for the statistical tests. The *p*-value for the hypothesis that the Gibbs method (or TEPITOPE) performs better than the LP method is estimated as the fraction of experiments where the Gibbs sampler (or TEPITOPE) has a better performance.

| **Original set** | **Neilsen**  **vs LP_top2** | **TEPITOPE vs**  **LP_top2** |  | **Homology reduced set** | **Neilsen vs LP_top2** | **TEPITOPE**  **vs**  **LP_top2** |
| --- | --- | --- | --- | --- | --- | --- |
| **set 1** | **0.032** | 0.866 |  | **set 1** | **0.009** | **0.005** |
| **set 2** | **0.034** | 0.346 |  | **set 2** | **0.004** | **0.038** |
| **set 3a** | **0.003** | 0.219 |  | **set 3a** | **0.000** | **0.005** |
| **set 3b** | **0.000** | 0.115 |  | **set 3b** | **0.001** | **0.019** |
| **set 4a** | **0.036** | 0.700 |  | **set 4a** | **0.040** | **0.009** |
| **set 4b** | 0.078 | 0.475 |  | **set 4b** | **0.008** | 0.054 |
| **set 5a** | **0.001** | 0.071 |  | **set 5a** | **0.000** | **0.042** |
| **set 5b** | **0.008** | **0.048** |  | **set 5b** | **0.007** | 0.063 |
| **geluk** | 0.058 | 0.570 |  | **geluk** | 0.102 | 0.503 |
| **southwood** | 0.644 | **0.010** |  | **southwood** | 0.611 | **0.010** |
